# Effect of induced hypoglycemia on inflammation and oxidative stress in type 2 diabetes and control subjects

**DOI:** 10.1038/s41598-020-61531-z

**Published:** 2020-03-16

**Authors:** Hassan Kahal, Anna Halama, Ahmed Aburima, Aditya M. Bhagwat, Alexandra E. Butler, Johannes Graumann, Karsten Suhre, Thozhukat Sathyapalan, Stephen L. Atkin

**Affiliations:** 1 Academic Endocrinology, Diabetes and Metabolism, Hull York Medical School, Hull, UK; 2 Centre for Cardiovascular and Metabolic Research, Hull York Medical School, Hull, UK; 3 Weill Cornell Medicine Qatar, Education City, PO, 24144 Doha, Qatar; 4 Diabetes Research Center (DRC), Qatar Biomedical Research Institute (QBRI), Hamad Bin Khalifa University (HBKU), Qatar Foundation (QF), PO Box, 34110 Doha, Qatar; 5 Proteomics Core, Weill Cornell Medicine-Qatar, Education City, PO Box, 24144 Doha, Qatar; 6 Scientific Service Group Biomolecular Mass Spectrometry, Max Planck Institute for Heart and Lung Research, Ludwigstr. 43, 61231 Bad Nauheim, Germany; 7 Royal College of Surgeon in Ireland, Manama, Bahrain

**Keywords:** Diabetes complications, Type 2 diabetes

## Abstract

Intensive diabetes control has been associated with increased mortality in type 2 diabetes (T2DM); this has been suggested to be due to increased hypoglycemia. We measured hypoglycemia-induced changes in endothelial parameters, oxidative stress markers and inflammation at baseline and after a 24-hour period in type 2 diabetic (T2DM) subjects versus age-matched controls. Case-control study: 10 T2DM and 8 control subjects. Blood glucose was reduced from 5 (90 mg/dl) to hypoglycemic levels of 2.8 mmol/L (50 mg/dl) for 1 hour by incremental hyperinsulinemic clamps using baseline and 24 hour samples. Measures of endothelial parameters, oxidative stress and inflammation at baseline and at 24-hours post hypoglycemia were performed: proteomic (Somalogic) analysis for inflammatory markers complemented by C-reactive protein (hsCRP) measurement, and proteomic markers and urinary isoprostanes for oxidative measures, together with endothelial function. Between baseline and 24 -hours after hypoglycemia, 15 of 140 inflammatory proteins differed in T2DM whilst only 1 of 140 differed in controls; all returned to baseline at 24-hours. However, elevated hsCRP levels were seen at 24-hours in T2DM (2.4 mg/L (1.2–5.4) vs. 3.9 mg/L (1.8–6.1), Baseline vs 24-hours, P < 0.05). In patients with T2DM, between baseline and 24-hour after hypoglycemia, only one of 15 oxidative stress proteins differed and this was not seen in controls. An increase (P = 0.016) from baseline (73.4 ng/mL) to 24 hours after hypoglycemia (91.7 ng/mL) was seen for urinary isoprostanes. Hypoglycemia resulted in inflammatory and oxidative stress markers being elevated in T2DM subjects but not controls 24-hours after the event.

## Introduction

While type 2 diabetes (T2DM) is associated with an increased risk of cardiovascular disease^1^, strict glycemic control does not result in obvious cardiovascular benefit in people with T2DM^2–4^. A link between strict glycemic control, hypoglycemia and increased cardiovascular morbidity and mortality has been observed in clinical studies^5,6^. Although the underlying mechanism remains unclear, increased inflammatory cytokines and a leukocytosis are reported after hypoglycemia^7,8^, suggesting a link between hypoglycemia and inflammation.

It is well recognized that oxidative stress leads to damage of proteins and deoxyribonucleic acid (DNA)^9^ and contributes to the diabetic complications of retinopathy, nephropathy, neuropathy and cardiovascular disorders^10–13^, and is directly linked to vascular inflammation, precipitating both endothelial cell dysfunction and vascular damage^14^. Oxidative stress results from excessive generation of free radicals and/or deficient defense mechanisms^15^, and leads to a disturbance of the physiological redox state^16^. The membrane associated nicotinamide dinucleotide phosphate oxidase enzyme complex is the primary source of reactive species in the vasculature, leading to superoxide generation^17,18^. Cytokines and hormones involved in the pathogenesis of vascular disease up-regulate vascular superoxide production^17^. The inflammation generated, in turn, can precipitate oxidative stress, further perpetuating the negative effects^19,20^. The endothelium is important in maintaining vascular homeostasis^21^ and endothelial dysfunction involves an impaired ability to regulate vascular tone together with anti-inflammatory and anticoagulatory alterations of the vasculature^22^. Moderate hypoglycemia has been shown to increase activation of prothrombotic and proinflammatory effects in type 1 diabetes^23^; therefore, in this study, we examined inflammatory markers, oxidative stress and endothelial function following iatrogenic hypoglycemia in subjects with and without T2DM^24^.

## Material and Methods

### Study design

A case-control study of T2DM subjects and age-matched controls. The study was approved by the Yorkshire and the Humber Research Ethics Committee and all study participants signed an informed consent form prior to participation. All institutional policies were followed for conductance of this study.

### Study participants

Healthy individuals and T2DM subjects were recruited from adverts placed in Hull Royal Infirmary, University of Hull, and in the local newspaper. Study inclusion criteria included both Caucasian men and women between 40 and 60 years of age who were non-smokers. Subjects in the normal control group had no chronic medical condition and were on no medications. Subjects in the T2DM group had had T2DM for less than 10 years with no history of microvascular disease (retinopathy, nephropathy or neuropathy), glycated hemoglobin (HbA1C) ≤9.5% (80.3 mmol/mol), and were treated with diet or oral medication only. All female patients were postmenopausal and were not taking any hormonal preparation. Exclusion criteria for both groups included, chronic medical conditions, use of anti-platelet medications, current smokers, evidence of ischemia on echocardiogram (ECG), a history of drop attacks, alcohol or drug abuse, psychiatric illness, or a history of seizures. No patients were on statin or antihypertensive therapy.

Subjects who fulfilled the study inclusion/exclusion criteria were screened, following a 10 hour fast, undergoing an ECG to rule out ischemia, a clinical examination, and routine blood tests to rule out anemia, hyperlipidemia, renal or hepatic impairment. Subjects in the control group had an oral glucose tolerance test to rule out diabetes. T2DM subjects had a diabetic foot examination to rule out diabetic neuropathy, a urine test for albumin/creatinine ratio to rule out diabetic nephropathy, and retinal screening (if this had not been performed within the last 6 months) to rule out diabetic retinopathy. All participants had an Endo-Pat test, as described below, to assess baseline endothelial function.

### Hyperinsulinemic euglycemic-hypoglycemic clamp studies

The clamp study was performed within 4 weeks of the screening visit when participants were asked to avoid exercise, caffeine and high antioxidant-containing food for 24 hours (assessed by food diary) prior to the procedure. Individuals with T2DM on oral medications were asked to stop their oral hypoglycemic agents two days prior to the visit. This visit was preceded by at least a 10 hour fast. Subjects provided an early morning urine sample.

The clamp procedure was performed as previously described^24^. The insulin infusion rate was constant throughout the clamp [60 mU/body surface area (m^2^)/min], while the rate of the 20% dextrose infusion was adjusted every 5 minutes to achieve the target blood glucose level. Body surface area (m^2^) was calculated as [0.007184 × (height(cm)^^^0.725) × (weight(kg)^^^0.425)]. Baseline glucose was 6.2 ± 1.1 mmol/l (112 ± 20 mg/dl) for T2DM that was reduced to 5.0 ± 0.5 mmol/l (90 ± 9 mg/dl) with the clamp for one hour, whilst the control group was maintained at 4.8 ± 0.5 mmol/l (86 ± 9 mg/dl) for one hour. The blood glucose level was dropped gradually to hypoglycemia, over 1 hour, to 2.8 mmol/L (50 mg/dl) and maintained at 2.8 mmol/L for 1 hour (hypoglycaemic clamp).

Twenty-four hours after the clamp study, in the morning immediately after and following a 10 hour fast, subjects provided an early morning urine sample, blood was taken and the Endo-Pat was performed.

### Biochemical markers

Urine samples were collected, and aliquots stored at −20 °C until batch analysis. Blood samples were separated immediately by centrifugation at 2000g for 15 minutes at 4 °C, and the aliquots were stored at −80 °C, within 30 minutes of blood collection, until batch analysis. High sensitivity C-reactive protein (hsCRP) was measured using Synchron systems CRPH reagent kit (Beckman-Coulter, UK) as per manufacturer’s protocol. Fasting plasma glucose (FPG) was measured using a Synchron LX 20 analyzer (Beckman-Coulter) according to the manufacturer’s recommended protocol. Total cholesterol, triglycerides, and high-density lipoprotein (HDL) cholesterol levels were measured enzymatically using a Synchron LX20 analyzer (Beckman-Coulter, High Wycombe, UK). Plasma metanephrine and normetanephrine were measured by tandem mass spectrometry. The between-run coefficients of variation (CV) for the metanephrine and normetanephrine measurements were 6.5–12.2% and 4.7–11.5%, respectively. Urinary isoprostane, 8-iso PGF_2α_, was measured by enzyme-linked immunosorbent assay (ELISA) using urinary isoprostane EIA kit (Oxford Biomedical Research, Oxford, USA) as per the manufacturer’s protocol, and by an operator who was blinded to study group of the participants^24^.

### Slow Off-rate Modified Aptamer (SOMA)-scan measurements

SOMAscan technology provides significant advantages in sample size, cost, time, multiplexing capability, dynamic range, and flexibility of readout over many alternate protein biomarker platforms. The protein quantification was performed using a Slow Off-rate Modified Aptamer (SOMAmer)–based protein array, as previously described^25,26^. Briefly, EDTA plasma samples were diluted and the following assay steps were performed: 1) binding – analytes and primer beads (PB)-SOMAmers (fully synthetic fluorophore-labeled SOMAmer coupled to a biotin moiety through a photocleavable linker) were equilibrated; 2) Catch 1 - all analyte/SOMAmers complexes were immobilized on a streptavidin-substituted support. Washing steps removed proteins not stably bound to PB-SOMAmers and bound protein was biotinylated; 3) Cleave - long-wave ultraviolet light was applied to release analyte-SOMAmer complexes into the solution; 4) Catch II – analyte-SOMAmer complexes were selectively immobilized on streptavidin support via the introduced analyte-borne biotinylation. Further washing was continued to select against unspecific analyte/SOMAmer complexes; 5) Elution – Denaturation caused disruption of analyte-SOMAmer complexes. Released SOMAmers serve as surrogates for quantification of analyte concentrations; 6) Quantification – hybridization to custom arrays of SOMAmer-complementary oligonucleotides.

Normalization of raw intensities, hybridization, median signal and calibration signal were performed based on the standard samples included on each plate, as previously described^27^.

We used version 3.1 of the SomaScan Assay, specifically targeting those proteins that have been linked with inflammation and oxidative stress in the SomaScan panel; at baseline, at the point of hypoglycemia and at 24 hours.

### Endo-Pat2000

Endo-PAT2000 was obtained from Itamar Medical Ltd, Israel. Endo-Pat was performed at baseline and at 24 hours following the clamp study. All tests were performed by the same operator, with the subject in the fasting state, and in a quiet and temperature-controlled room (23 °C) with the participant relaxed in a semi-recumbent position. Probes were placed on the index finger of each arm to measure peripheral arterial tone (PAT). PAT was recorded in both arms for the duration of the test. After 5 minutes of rest (baseline), the blood pressure cuff was inflated to 220–230 mmHg to stop the blood supply in the study arm for 5 minutes, and then deflated to release the blood supply for a further 5 minutes. A reactive hyperemic index (RHI) is a measure of the change in PAT in the study arm after occlusion compared to baseline, and adjusted for changes in the control arm. Bonetti *et al*.^28^ reported that a measurement of RHI lower than the cut-off value of 1.67 provides a sensitivity of 82% and a specificity of 77% in diagnosing coronary endothelial dysfunction. Endo-PAT reproducibility was within acceptable limits, ICC = 0.74, and coefficient of variation 12 ± 2.2%^29^.

### Statistical analysis

This was a discovery study and no power analysis could be undertaken given that no similar studies have been undertaken. Data trends were visually evaluated for each parameter and non-parametric tests were applied on data that violated the assumptions of normality when tested using the Kolmogorov-Smirnov Test. Within group comparisons are as follows: changes from baseline at each stage (euglycemia, hypoglycemia and 24 hours) were compared using the paired t-test (or Wilcoxon signed-rank test for non-normally distributed data). Repeated measures were compared by analysis of variance (ANOVA); non-normally distributed data were log-transformed. The sample size was too small to adjust for baseline covariates. A two tailed p value of <0.05 was considered statistically significant. ANOVA post-hoc comparisons were adjusted using the Sidak test, to correct for multiple comparisons. Statistical analysis was performed using the Predictive Analytics Software (PASW) statistics 25 package (Statistical Package for the Social Sciences (SPSS) Inc., Chicago, USA)^24^.

For the proteomic analysis we fitted an intercept-free general linear model as a function of subgroup (i.e. condition:timepoint), while taking the patient identification (ID) as a random effect using the R package limma. Subsequently, we computed the p value for two contrasts: baseline to hypoglycemia for both T2DM and controls, and false discovery rate (FDR) corrected at a value of <0.05 as the cutoff for significance.

### Ethics approval and consent to participate

The study was approved by the Yorkshire and the Humber Research Ethics Committee and all study participants signed an informed consent form prior to participation.

### Consent for publication

All authors gave their consent for publication.

## Results

18 participants were recruited (10 people with T2DM, 8 controls). In the T2DM group, the median duration of diabetes was 10 months (range 5–24 months); seven participants (70%) were on metformin therapy, while three were diet controlled. Participants’ demographics and baseline characteristics are summarized in Table 1.

**Table 1:** Demographic and baseline characteristics of study participants.

	T2D (n = 10)	Controls (n = 8)
Age (years)	47.0 (42.0–51.5)	47.5 (40.8–52.8)
Males (%)	8 (80%)	5 (62.5%)
Weight (kg)	103.1 (87.0–109.1)	85.5 (71.7–99.2)
BMI (kg/m^2^)	35.8 (27.3–40.9)	28.2 (24.2–32.8)*
Waist circumference (cm)	117.0 (99.5–124.7)	91.0 (82.9–111.3)*
Hip circumference (cm)	113.9 (105.8–128.2)	103.0 (99.5–113.7)
Waist/hip	0.99 (0.91–1.1)	0.90 (0.83–0.98)
Systolic BP (mmHg)	132 (111–142)	123 (116–132)
Diastolic BP (mmHg)	74 (68–85)	76 (69–82)
Cholesterol (mmol/L)	5.3 (4.4–5.7)	5.6 (4.2–5.7)
Triglycerides (mmol/L)	1.3 (0.98–2.2)	1.3 (0.78–1.5)
HDL (mmol/L)	1.2 (0.98–1.4)	1.3 (1.2–1.3)
LDL (mmol/L)	3.2 (2.8–3.7)	3.5 (2.7–3.7)
Chol/HDL	3.9 (3.6–5.8)	4.0 (3.4–4.3)
HbA1C (mmol/mol)	45.5 (39–56.3)	34 (31–36)*
HbA1C (%)	6.3 (5.7–7.3)	5.3 (5.0–5.4)*

Data presented as median (25th/75th centiles). Waist/hip, waist to hip ratio; BP, blood pressure; HDL, high density lipoprotein; LDL, low density lipoprotein; Chol/HDL, cholesterol to HDL ratio; HbA1C, hemoglobin A1C. *P < 0.05.

### Biochemical markers

**Figure 1:**
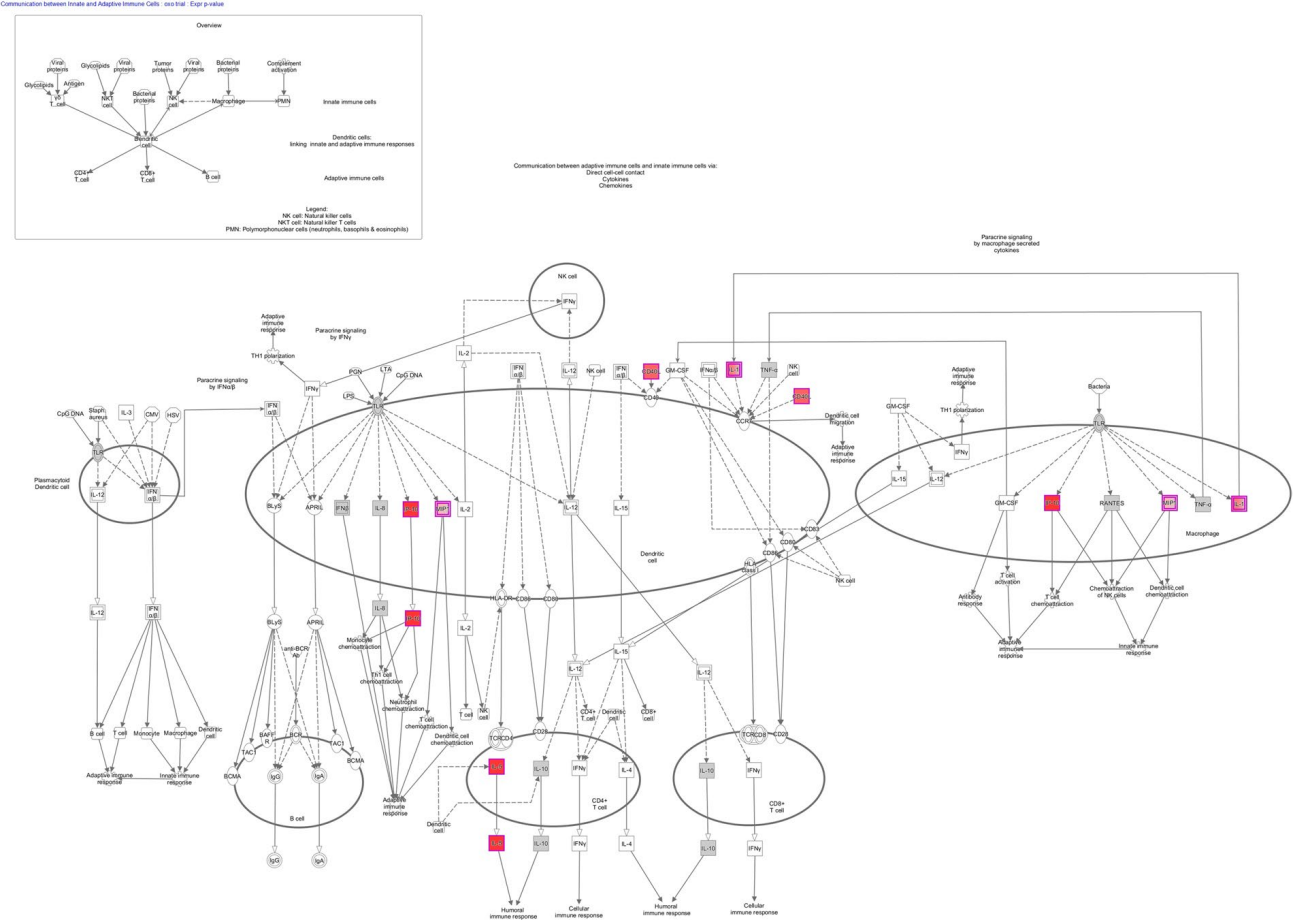
Ingenuity Pathway Analysis showing communication between adaptive and innate immune cells via direct cell-to-cell contact, cytokines and chemokines.

For the inflammatory marker panel, comparison of baseline to hypoglycemia in T2DM subjects showed 15 proteins of 140 that were significantly different (Table 2) after multiple comparison testing: C-X-C motif chemokine 10 (CXCL10), Interleukin-5 (IL5), Azurocidin (AZU1), C-type lectin domain family 7 member A (CLEC7A), Serine/threonine-protein kinase (TBK1), Protein kinase C zeta type (PRKCZ), Ribosomal protein S6 kinase alpha-5 (RPS6KA5), CD40 ligand (CD40LG), Interleukin-34 (IL34), High mobility group protein B1 (HMGB1), Protein S100-A9 (S100A9), Interleukin-1 beta (IL1B), C-C motif chemokine 19 (CCL19), Sialoadhesin (SIGLEC1) and Interleukin 10 receptor beta subunit (IL10RB). Comparison of T2DM and controls revealed that 4 proteins remained significantly different (Table 2) after multiple comparison testing: Prostaglandin G/H synthase 2 (PTGS2), HMGB1, IL5 and CXCL10. Ingenuity Pathway Analysis showing communication between adaptive and innate immune cells via direct cell-to-cell contact, cytokines and chemokines is shown in Fig. 1.

**Table 2:** Inflammatory stress protein panel in patients with type 2 diabetes from baseline following hypoglycemia showing the 15 of 140 proteins that were significantly altered with a false discovery rate (fdr) of <0.05; the “beta” indicates the beta coefficient and reflects the direction of change.

Protein	UniProt	T2D		Control	
		beta	p-value	beta	p-value
Interleukin-5	P05113	0.39	2.78*10^−04^	0.20	1.30*10^−01^
Azurocidin	P20160	0.47	5.75*10^−04^	−0.25	1.32*10^−01^
Ribosomal protein S6 kinase alpha-5	O75582	0.44	1.75*10^−03^	−0.24	1.62*10^−01^
CD40 ligand	P29965	0.49	1.75*10^−03^	−0.36	5.64*10^−02^
High mobility group protein B1	P09429	0.39	2.20*10^−03^	0.39	1.25*10^−02^
Interleukin-34	Q6ZMJ4	−0.19	1.99*10^−03^	0.12	1.00*10^−01^
C-X-C motif chemokine 10	P02778	−0.54	2.61*10^−04^	−0.22	2.19*10^−01^
Protein S100-A9	P06702	0.52	2.82*10^−03^	0.23	2.81*10^−01^
C-C motif chemokine 19	Q99731	−0.33	4.16*10^−03^	−0.16	2.51*10^−01^
Sialoadhesin	Q9BZZ2	−0.18	4.26*10^−03^	−0.07	3.80*10^−01^
Serine/threonine-protein kinase TBK1	Q9UHD2	0.25	1.20*10^−03^	−0.07	4.42*10^−01^
C-type lectin domain family 7 member A	Q9BXN2	0.55	7.59*10^−04^	0.13	5.11*10^−01^
Protein kinase C zeta type	Q05513	0.29	1.71*10^−03^	0.06	5.76*10^−01^
Interleukin-1 beta	P01584	0.70	4.13*10^−03^	−0.17	5.77*10^−01^
Interleukin-10 receptor subunit beta	Q08334	−0.16	4.67*10^−03^	0.03	6.24*10^−01^
Prostaglandin G/H synthase 2	P35354	0.15	6.73*10^−02^	0.44	5.33*10^−05^
Fibroblast growth factor 8 isoform A	P55075	0.56	1.34*10^−03^	−0.25	2.10*10^−01^

Details of the panel of all of the inflammatory proteins measured are shown in Supplementary Tables 1 and 2 for diabetes patients and controls, respectively; it can be seen therein that other proteins were showing a trend of deregulation, but were not significantly different, likely due to the small sample size. A comparison of baseline to 24 hours showed no difference in any of the SomaScan proteins identified.

In both control and T2DM subjects, the inflammatory marker hsCRP increased maximally at 24 hours after induction of hypoglycemia, but the increase was significantly relevant only in the T2DM group (p < 0.01) (Table 3).

**Table 3:** A comparison of biochemical, inflammatory and endothelial function markers during the insulin clamp.

Group	Sample	Baseline	Euglycaemia	Hypoglycaemia	24 hours	Comments
T2D	Metanephrine (80–510) pmol/L	86 (42–122)	121 (71–147)	284 (199–308)^	101 (58–128)	ANOVA (P = 0.001)
	Normetanephrine (120–1180) pmol/L	153 (127–223)	143.5 (81–262)	157 (128–282)	202 (133–232)	ANOVA (P = 0.9)
	hsCRP(0–8) mg/L	2.4 (1.2–5.4)	2.2 (1.6–6)*	2.1 (1.1–5.1)	3.9 (1.8–6.1)^	ANOVA (P = 0.01)
	RHI	2.0 (1.7–2.7)			2.7 (2.0–3.2)	P = 0.13
	Urinary isoprostanes (ng/ml)	73.4 (58.1–96.5)			91.7 (76.1–97.0)	P = 0.016
Controls	Metanephrine (80–510) pmol/L	153 (88–225)	150 (68–222)	415 (250–607)^^	107 (66–183)	ANOVA (P = 0.048)
	Normetanephrine (120–1180) pmol/L	234 (157–324)	225 (183–241)	225 (154–358)	231 (177–301)	ANOVA (P = 0.17)
	hsCRP(0–8) mg/L	1.2 (0.9–2.3)	0.9 (0.4–2.4)	1.0 (0.7–2.4)	1.6 (1.0–2.7)^^^	ANOVA (P = 0.045)
	RHI	2.0 (1.7–2.6)			2.1 (1.8–2.5)	P = 0.80
	Urinary isoprostanes (ng/ml)	53.7 (11.2–79.7)			85.0 (8.9–95.2)	P = 0.28

Data presented as median (25th/75th centiles). T2DM, type 2 diabetes mellitus; hsCRP, high sensitivity C-reactive protein; RHI, reactive hyperemic index. All significant P values are highlighted. *P < 0.05 compared to baseline and 24 hours. ^P < 0.05 compared to any other time point. ^P < 0.05 compared to euglycemia and 24 hours. ^^^P < 0.05 compared to baseline and hypoglycemia. All post-hoc comparisons are Sidak test adjusted.

RHI = Reactive hyperemia index for endothelial function. CRP = C reactive protein. *P < 0.05 compared to baseline and 24 hours. ^P < 0.05 compared to any other time point. ^^P < 0.05 compared to euglycaemia and 24 hours. ^^^P < 0.05 compared to baseline and hypoglycaemia. All post-hoc comparisons are Sidak test adjusted.

A subgroup analysis on males and females alone did not show significant differences between the genders and therefore the results were pooled.

In the oxidative stress marker panel, only PTGS2 differed between T2DM and controls (p < 0.0001), and comparison of baseline to hypoglycemia in T2DM subjects showed fibroblast growth factor 8 (FGF8) (p < 0.0001) was significantly different, after multiple testing correction.

Whilst isoprostane (an oxidative stress marker) levels increased in both groups at 24 hours post clamp, only in T2DM were they significantly different from baseline (p < 0.05) (Table 3).

In both T2DM and control groups, plasma metanephrine significantly increased during the hypoglycaemic clamp and returned to baseline after 24 hours (Table 3), though between group changes did not differ. There was no significant change in plasma normetanephrine during the study in either group.

A subgroup analysis on males and females alone did not show significant differences between the genders and therefore the results were pooled.

### Endothelial function

There was no significant change, in either group, in RHI at 24 hours compared to baseline (Table 3).

## Discussion

Inflammatory regulators were increased at the time of hypoglycemia and, despite returning to normal at 24 hours, there was a residual effect on inflammation as seen by the increase in the inflammatory marker CRP. In line with the study objective, we found that these inflammatory changes were exaggerated in T2DM in comparison with the controls that showed a significant increase in only one inflammatory protein in contrast to T2DM where 15 significantly altered proteins were identified. Additionally, CRP was elevated at 24 hours in T2DM but not in controls. Others have reported an increase in inflammation at the time of hypoglycemia^7,8,30^ and with an increase in both prothrombotic and proinflammatory parameters in type 1 diabetes^23^, but not in such a comprehensive manner looking at a broad proteomic approach of inflammatory proteins. Residual inflammation with an elevated CRP at 24 hours has not previously been reported in people with T2DM. This suggests that the enhanced basal inflammation recognized in T2DM has an exaggerated effect in response to hypoglycemia that is persistent because, although the proteomic markers had returned to baseline at 24 hours, no time course was done after the hypoglycemic event to determine precisely when inflammation returned to baseline.

The majority of the inflammatory proteins that were found to be significantly elevated have been associated with increased cardiovascular disease such as, interleukin-5, cluster of differentiation 40 (CD40) and interleukin-1beta^31^, whilst FGF8 has been more associated with vascular remodeling.

Amongst the oxidative stress markers, only FGF8 was elevated and differentiated T2DM from controls. The elevation in FGF8 may suggest activation of protective mechanisms as FGF8 has been shown to inhibit oxidative stress^32^ and to protect neurons from oxidative stress^33^ in an *in vitro* setting. A residual effect on oxidative stress, with an elevation in urinary isoprostanes after 24 hours, was evident in T2DM subjects but not in non-diabetic controls. The urine collection at 24 hours was a spot urine that would reflect overnight urine production and was not collected at the time of the hypoglycemic event, suggesting that the induced oxidative stress continued for a period after the event. It is unclear if an exaggerated inflammatory response with more inflammatory markers resulted in enhanced oxidative stress, or if, conversely, the oxidative stress led to inflammation, or if indeed the result was an additive effect of both; more time points over the 24 hours period would be needed to determine this.

Oxidative stress is directly linked to vascular inflammation, precipitating both endothelial cell dysfunction and vascular damage^14^. There were no changes in endothelial function seen in this study at 24 hours; however, it was not possible technically to undertake the Endo-Pat at the time of hypoglycemia concomitantly with the insulin clamp, and changes at this time point may have been missed. This result differs from a report using flow mediated vasodilation^7^ wherein endothelial function was reduced following hypoglycemia but was, in fact, blunted by repeated hypoglycemia, raising the question of whether clamping the hypoglycemia for one hour was responsible for this difference.

The Ingenuity Pathway Analysis (IPA) of the inflammatory proteins showed enrichment in the canonical pathways associated with communication between innate and adaptive immune cells (Supplementary Table 2). The innate immune response resulting in inflammation depends upon recognition of pattern-recognition receptors (PRRs). PRRs are expressed mainly in immune and inflammatory cells such as monocytes, neutrophils and antigen-presenting cells (APCs) including macrophages and dendritic cells^34,35^. The association shown in the ingenuity pathway suggests that activation of PRRs resulted, leading to the induction of the inflammatory response with proinflammatory cytokines and type I interferons (interferon-α and interferon-β)^36^. These data are in accord with the proinflammatory effects reported following induced hypoglycemia with mobilization of the leukocyte response and induction of proinflammatory changes in immune cells that may promote a sustained inflammatory state^37^.

It is well recognized that inflammation is associated with adverse cardiac events and elevated levels of CRP, and other inflammatory biomarkers have been associated with multiple cardiovascular endpoints^38^ as well as sudden cardiac death^39^. An increase in oxidative stress leading to the overproduction of reactive oxygen species, which is often toxic to cells, causing damage to all components of the cells, such as proteins, DNA, and lipids^20^. Oxidative stress is known to be a major injury mechanism implicated in the pathogenesis of disease progression including ischemic myocardial injury, and may be caused by, or result in, inflammation^14^. In a population study, an increase in proinflammatory markers was related to hypoglycemia but not to macrovascular complications^40^. Our data suggest that the adverse effects of hypoglycemia on both inflammation and oxidative stress may persist well beyond the normalization of blood glucose levels.

In a recent large observational study in T2DM subjects^41^, strict glycemic control, with a median HbA1C of 6.4%, was associated with increased all-cause as well as cardiovascular mortality. In addition, in the three large trials: Action to Control Cardiovascular Risk in Diabetes (ACCORD)^3^, Action in Diabetes and Vascular Disease: Preterax and Diamicron MR Controlled Evaluation (ADVANCE)^2^ and Veterans Affairs Diabetes Trial (VADT)^4^, which failed to show cardiovascular benefit with strict glycemic control in T2DM subjects, participants in the intensive treatment arm had significantly more episodes of severe hypoglycemia. Hypoglycemia was not thought to explain the increased mortality rate in the intensive treatment arm of the ACCORD study^42^ though no accountable cause was identified^42–45^. However, a link between hypoglycemia and increased mortality may exist, especially as episodes of hypoglycemia might pass unrecognized and self-reporting of hypoglycemia is known to be inaccurate. Furthermore, a cardiac event may not be linked to an episode of hypoglycemia occurring 24 hours previously. Enhanced inflammation and increased oxidative stress may contribute to the underlying mechanism of the association of hypoglycemia with subsequent cardiac death.

An increase in metanephrine levels was expected with hypoglycaemia in both groups as we have shown before^46^ indicative of the physiological response; however, there was the suggestion that the response was blunted in T2DM, though there was no statistical difference between groups.

The strengths of this study include the inclusion of a group of T2DM subjects who were relatively treatment naïve and not on poly-pharmacy, and an age-matched healthy control group. The main study limitation is the small study numbers and, with a larger population, the robust response to the number of inflammatory proteins increased by hypoglycemia may have been greater. Those with T2DM were more obese than the controls and it cannot be excluded that obesity may have contributed to the enhanced inflammatory response. The treatment of those with T2DM may have altered the inflammatory responses. The changes that were looked for were at 24 hours and whilst the changes in oxidative stress and inflammation in T2DM were clearly significant at 24 hours, there were no changes in endothelial function that may have arisen prior to this; however, this does still raise many questions about the impact of hypoglycemia on cardiovascular events that may occur 24 hours later in this group of people with high cardiovascular risk. The choice of the patients with T2DM were those on diet alone and on a stable dose of metformin and it is unknown if a different oxidative or inflammatory response would be found in those patients on a sulphonylurea or on insulin therapy.

## Conclusion

In conclusion, induced hypoglycemia significantly increased markers of inflammation and a marker of oxidative stress at the point of hypoglycemia, with increased integrative markers of both inflammation and oxidative stress at 24 hours following the hypoglycemic episode in T2DM subjects but not controls. This showed an increased inflammatory and oxidative stress response to hypoglycemia in T2DM that may be additive and generalizable to non-iatrogenically induced hypoglycemic episodes in subjects with T2DM, though endothelial function was unaffected.

## Supplementary information

Supplementary information.

## Data Availability

All the data for this study will be made available upon reasonable request to the corresponding author.
