# Author Correction: Effect of induced hypoglycemia on inflammation and oxidative stress in type 2 diabetes and control subjects

**DOI:** 10.1038/s41598-020-66189-1

**Published:** 2020-06-19

**Authors:** Hassan Kahal, Anna Halama, Ahmed Aburima, Aditya M. Bhagwat, Alexandra E. Butler, Johannes Graumann, Karsten Suhre, Thozhukat Sathyapalan, Stephen L. Atkin

**Affiliations:** 1 Academic Endocrinology, Diabetes and Metabolism, Hull York Medical School, Hull, UK; 2 Centre for Cardiovascular and Metabolic Research, Hull York Medical School, Hull, UK; 3 Weill Cornell Medicine Qatar, Education City, PO, 24144 Doha, Qatar; 4 Diabetes Research Center (DRC), Qatar Biomedical Research Institute (QBRI), Hamad Bin Khalifa University (HBKU), Qatar Foundation (QF), PO Box, 34110 Doha, Qatar; 5 Proteomics Core, Weill Cornell Medicine-Qatar, Education City, PO Box, 24144 Doha, Qatar; 6 Scientific Service Group Biomolecular Mass Spectrometry, Max Planck Institute for Heart and Lung Research, Ludwigstr. 43, 61231 Bad Nauheim, Germany; 7 Royal College of Surgeon in Ireland, Manama, Bahrain

Correction to: *Scientific Reports* 10.1038/s41598-020-61531-z, published online 16 March 2020

The original version of this Article contained a typographical error in the spelling of the author Johannes Graumann, which was incorrectly given as Johannes Grauman. This has now been corrected in the PDF and HTML versions of the Article, and in the accompanying Supplementary Information file.

