# Supplementary material for: Effect of induced hypoglycemia on inflammation and oxidative stress in type 2 diabetes and control subjects
Source: Sci Rep. 2020 Mar 16;10:4750. doi: 10.1038/s41598-020-61531-z (PMC7075968; doi:10.1038/s41598-020-61531-z)
Supplement: Supplementary file 1 — Supplementary information. [file 41598_2020_61531_MOESM1_ESM.pdf]

Effect of induced hypoglycemia on inflammation and oxidative stress in type 2 diabetes and control subjects

Hassan Kahal PhD[1,2*], Anna Halama, PhD[3]*, Ahmed Aburima PhD[2], Aditya M. Bhagwat PhD[3], Alexandra E. Butler MD[4#], Johannes Graumann, PhD[5,6], Karsten Suhre, PhD[5], Thozhukat Sathyapalan MD[1], Stephen L Atkin MD[7]

1. Academic Endocrinology, Diabetes and Metabolism, Hull York Medical School, Hull, UK.

2. Centre for Cardiovascular and Metabolic Research, Hull York Medical School, Hull, UK.

3. Weill Cornell Medicine Qatar, Education City, PO 24144 Doha, Qatar.

4. Diabetes Research Center (DRC), Qatar Biomedical Research Institute (QBRI), Hamad Bin Khalifa University (HBKU), Qatar Foundation (QF), PO Box 34110, Doha, Qatar

5. Proteomics Core, Weill Cornell Medicine-Qatar, Education City, PO Box 24144, Doha, Qatar

6. Scientific Service Group Biomolecular Mass Spectrometry, Max Planck Institute for Heart and Lung Research, Ludwigstr. 43, 61231 Bad Nauheim Germany

7. Royal College of Surgeon in Ireland, Bahrain.

* These authors contributed equally to this work.

Supplementary Table 1. Inflammatory protein panel for all proteins for the type 2 diabetes patients, p value and fdr values (<0.05 for fdr was considered significant)

Target Full Name	P	fd
C-X-C motif chemokine 10	0.0003	0.0196
Interleukin-5	0.0003	0.0196
Azurocidin	0.0006	0.0268
C-type lectin domain family 7 member A	0.0008	0.0268
Serine/threonine-protein kinase TBK1	0.0012	0.0308
Protein kinase C zeta type	0.0017	0.0308
Ribosomal protein S6 kinase alpha-5	0.0017	0.0308
CD40 ligand	0.0017	0.0308
Interleukin-34	0.0020	0.0311
High mobility group protein B1	0.0022	0.0311
Protein S100-A9	0.0028	0.0361
Interleukin-1 beta	0.0041	0.0430
C-C motif chemokine 19	0.0042	0.0430
Sialoadhesin	0.0043	0.0430
Interleukin-10 receptor subunit beta	0.0047	0.0439
Fractalkine	0.0084	0.0745
Complement C3b, inactivated	0.0091	0.0754
C-X-C motif chemokine 5	0.0109	0.0851
Protein DJ-1	0.0115	0.0851
Tumor necrosis factor receptor superfamily member 11A	0.0147	0.1034
C-C motif chemokine 7	0.0175	0.1174
Interferon alpha-2	0.0249	0.1589
Tumor necrosis factor receptor superfamily member 19L	0.0259	0.1589
Lymphotactin	0.0297	0.1744
C-C motif chemokine 20	0.0333	0.1802
Tumor necrosis factor receptor superfamily member 21	0.0346	0.1802
Tumor necrosis factor receptor superfamily member 11B	0.0352	0.1802
Toll-like receptor 4:Lymphocyte antigen 96 complex	0.0366	0.1802
Interleukin-37	0.0371	0.1802
C-C motif chemokine 1	0.0383	0.1802
Interleukin-17B	0.0411	0.1869
C-C motif chemokine 15	0.0494	0.2178
C-C motif chemokine 4-like	0.0516	0.2206
Interleukin-23 receptor	0.0532	0.2206
Transforming growth factor beta-1	0.0623	0.2448
Growth-regulated alpha protein	0.0662	0.2448
Advanced glycosylation end product-specific receptor, soluble	0.0663	0.2448

Prostaglandin G/H synthase 2	0.0673	0.2448
Toll-like receptor 2	0.0677	0.2448
Oxidized low-density lipoprotein receptor 1	0.0718	0.2530
Insulin-like growth factor-binding protein 4	0.0797	0.2741
C-C motif chemokine 3-like 1	0.0908	0.3050
Phosphatidylinositol 4,5-bisphosphate 3-kinase catalytic subunit alpha isoform:Phosphatidylinositol 3-kinase regulatory subunit alpha complex	0.0946	0.3103
Tumor necrosis factor receptor superfamily member 1B	0.0969	0.3106
Interleukin-17D	0.1235	0.3793
Lymphocyte antigen 86	0.1264	0.3793
EGF-like module-containing mucin-like hormone receptor-like 2	0.1264	0.3793
Interleukin-6	0.1468	0.4313
Eotaxin	0.1678	0.4758
Endothelial monocyte-activating polypeptide 2	0.1687	0.4758
Interleukin-2 receptor subunit alpha	0.1725	0.4770
Interleukin-23	0.1831	0.4964
Tumor necrosis factor receptor superfamily member 1A	0.1970	0.5160
C-C motif chemokine 24	0.1976	0.5160
C-C motif chemokine 3	0.2030	0.5204
Tumor necrosis factor receptor superfamily member 9	0.2126	0.5354
Tumor necrosis factor receptor superfamily member 8	0.2183	0.5400
Interleukin-1 receptor-like 2	0.2284	0.5554
Hepatitis A virus cellular receptor 2	0.2374	0.5628
Interleukin-22	0.2431	0.5628
Lysozyme C	0.2479	0.5628
CD5 antigen-like	0.2504	0.5628
C-reactive protein	0.2515	0.5628
C-C motif chemokine 14	0.2578	0.5680
C3a anaphylatoxin des Arginine	0.2656	0.5687
Ras-related C3 botulinum toxin substrate 1	0.2684	0.5687
C5a anaphylatoxin	0.2702	0.5687
P-Selectin	0.2751	0.5704
Interleukin-10	0.2812	0.5747
C-C motif chemokine 13	0.2913	0.5789
Ck-beta-8-1	0.2927	0.5789
Complement C3	0.2956	0.5789
C-C motif chemokine 18	0.3089	0.5921
Thrombospondin-1	0.3167	0.5921
Interleukin-17F	0.3234	0.5921
Tumor necrosis factor receptor superfamily member 18	0.3245	0.5921
Interleukin-8	0.3252	0.5921
C3a anaphylatoxin	0.3276	0.5921
Complement C3b	0.3357	0.5921

C-C motif chemokine 2	0.3359	0.5921
Kininogen-1	0.3491	0.6077
Tyrosine-protein kinase HCK	0.3582	0.6160
Complement C3d fragment	0.3739	0.6313
C-X-C motif chemokine 6	0.3761	0.6313
Protein kinase C theta type	0.3962	0.6511
C-C motif chemokine 21	0.4014	0.6511
C-C motif chemokine 25	0.4054	0.6511
MAP kinase-activated protein kinase 2	0.4118	0.6511
Interleukin-27	0.4153	0.6511
C-C motif chemokine 17	0.4192	0.6511
C-X-C motif chemokine 13	0.4202	0.6511
C-C motif chemokine 22	0.4628	0.7030
Interleukin-13	0.4637	0.7030
PSA:alpha-1-antichymotrypsin complex	0.4750	0.7121
Tumor necrosis factor	0.4828	0.7121
C-X-C motif chemokine 11	0.4850	0.7121
Bone morphogenetic protein 6	0.4936	0.7121
Neutrophil-activating peptide 2	0.4961	0.7121
C-C motif chemokine 8	0.5000	0.7121
Tumor necrosis factor receptor superfamily member 4	0.5145	0.7228
Retinoic acid receptor responder protein 2	0.5177	0.7228
Peroxiredoxin-5, mitochondrial	0.5280	0.7270
Interleukin-17A	0.5351	0.7270
Tumor necrosis factor receptor superfamily member 10A	0.5375	0.7270
Macrophage colony-stimulating factor 1	0.5414	0.7270
Tumor necrosis factor receptor superfamily member 14	0.5558	0.7364
Alpha-1-antichymotrypsin	0.5642	0.7364
CD27 antigen	0.5660	0.7364
Macrophage colony-stimulating factor 1 receptor	0.5735	0.7364
Phosphatidylinositol 4,5-bisphosphate 3-kinase catalytic subunit gamma isoform	0.5791	0.7364
Calcium/calmodulin-dependent protein kinase type 1D	0.5830	0.7364
Connective tissue-activating peptide III	0.5850	0.7364
Natural cytotoxicity triggering receptor 3	0.5961	0.7396
Tumor necrosis factor receptor superfamily member 3	0.5980	0.7396
C-C motif chemokine 23	0.6254	0.7618
Complement C5b-C6 complex	0.6267	0.7618
Platelet factor 4	0.6351	0.7653
CD97 antigen	0.6429	0.7683
E-Selectin	0.6499	0.7701
Mast/stem cell growth factor receptor Kit	0.6754	0.7879
Complement C4b	0.6762	0.7879

Macrophage migration inhibitory factor	0.6930	0.8009
Annexin A1	0.7091	0.8129
Interleukin-1 alpha	0.7208	0.8196
Complement C4	0.7453	0.8406
C-C motif chemokine 16	0.7713	0.8631
Interleukin-1 Receptor accessory protein	0.7827	0.8690
Interleukin-18 receptor accessory protein	0.8035	0.8771
Complement C5	0.8075	0.8771
Tyrosine-protein kinase Lyn, isoform B	0.8087	0.8771
Allograft inflammatory factor 1	0.8225	0.8811
Sphingosine kinase 1	0.8249	0.8811
Extracellular matrix protein 1	0.8390	0.8895
Tumor necrosis factor receptor superfamily member 25	0.8612	0.9062
Tumor necrosis factor ligand superfamily member 4	0.8930	0.9327
Tumor necrosis factor receptor superfamily member 6B	0.9051	0.9370
Carbohydrate sulfotransferase 2	0.9104	0.9370
C-C motif chemokine 5	0.9285	0.9466
Tyrosine-protein kinase Lyn	0.9331	0.9466
Group IIE secretory phospholipase A2	0.9576	0.9644
Tumor necrosis factor-inducible gene 6 protein	0.9710	0.9710

Supplementary Table 2. Inflammatory protein panel for all proteins for the control subjects, p value and fdr values (<0.05 for fdr was considered significant)

Target Full Name	p	fdr
Prostaglandin G/H synthase 2	5.3E-05	7.5E-03
High mobility group protein B1	1.3E-02	7.9E-01
Tumor necrosis factor receptor superfamily member 8	3.0E-02	7.9E-01
Tumor necrosis factor receptor superfamily member 11B	3.6E-02	7.9E-01
Interferon alpha-2	4.2E-02	7.9E-01
C-C motif chemokine 17	4.9E-02	7.9E-01
CD40 ligand	5.6E-02	7.9E-01
Platelet factor 4	6.1E-02	7.9E-01
Interleukin-37	6.1E-02	7.9E-01
C-C motif chemokine 21	8.1E-02	7.9E-01
CD97 antigen	8.5E-02	7.9E-01
C-C motif chemokine 15	9.5E-02	7.9E-01
Interleukin-34	1.0E-01	7.9E-01
Interleukin-6	1.0E-01	7.9E-01
Tumor necrosis factor receptor superfamily member 14	1.2E-01	7.9E-01
Lymphotactin	1.2E-01	7.9E-01
Ras-related C3 botulinum toxin substrate 1	1.2E-01	7.9E-01
C-C motif chemokine 1	1.2E-01	7.9E-01
Tumor necrosis factor receptor superfamily member 18	1.2E-01	7.9E-01
Connective tissue-activating peptide III	1.3E-01	7.9E-01
Sphingosine kinase 1	1.3E-01	7.9E-01
Interleukin-5	1.3E-01	7.9E-01
Azurocidin	1.3E-01	7.9E-01
Complement C4b	1.4E-01	7.9E-01
Interleukin-10	1.4E-01	7.9E-01
C-C motif chemokine 3-like 1	1.5E-01	7.9E-01
C-C motif chemokine 5	1.6E-01	7.9E-01
Ribosomal protein S6 kinase alpha-5	1.6E-01	7.9E-01
MAP kinase-activated protein kinase 2	1.7E-01	7.9E-01
C-C motif chemokine 20	1.7E-01	7.9E-01
Tyrosine-protein kinase Lyn, isoform B	1.9E-01	7.9E-01
C-C motif chemokine 2	1.9E-01	7.9E-01
Alpha-1-antichymotrypsin	1.9E-01	7.9E-01
Growth-regulated alpha protein	2.0E-01	7.9E-01
Neutrophil-activating peptide 2	2.0E-01	7.9E-01
Retinoic acid receptor responder protein 2	2.1E-01	7.9E-01
Fractalkine	2.1E-01	7.9E-01

Thrombospondin-1	2.1E-01	7.9E-01
C-X-C motif chemokine 10	2.2E-01	7.9E-01
C-C motif chemokine 4-like	2.2E-01	7.9E-01
Tyrosine-protein kinase Lyn	2.4E-01	8.1E-01
C-C motif chemokine 19	2.5E-01	8.2E-01
C-C motif chemokine 8	2.5E-01	8.2E-01
Interleukin-17F	2.6E-01	8.2E-01
Protein S100-A9	2.8E-01	8.2E-01
Kininogen-1	2.8E-01	8.2E-01
Extracellular matrix protein 1	2.8E-01	8.2E-01
C3a anaphylatoxin	2.9E-01	8.2E-01
Endothelial monocyte-activating polypeptide 2	2.9E-01	8.2E-01
Tumor necrosis factor receptor superfamily member 3	2.9E-01	8.2E-01
C3a anaphylatoxin des Arginine	3.0E-01	8.2E-01
Complement C4	3.0E-01	8.2E-01
Toll-like receptor 4:Lymphocyte antigen 96 complex	3.1E-01	8.2E-01
Complement C5b-C6 complex	3.3E-01	8.6E-01
Macrophage colony-stimulating factor 1 receptor	3.4E-01	8.6E-01
Tumor necrosis factor receptor superfamily member 21	3.4E-01	8.6E-01
Protein kinase C theta type	3.6E-01	8.7E-01
C-C motif chemokine 24	3.6E-01	8.7E-01
Tumor necrosis factor receptor superfamily member 11A	3.6E-01	8.7E-01
Tyrosine-protein kinase HCK	3.8E-01	8.8E-01
Sialoadhesin	3.8E-01	8.8E-01
Eotaxin	3.9E-01	8.8E-01
C-X-C motif chemokine 6	3.9E-01	8.8E-01
CD27 antigen	4.1E-01	9.0E-01
Tumor necrosis factor receptor superfamily member 1A	4.2E-01	9.0E-01
C-reactive protein	4.2E-01	9.0E-01
Serine/threonine-protein kinase TBK1	4.4E-01	9.3E-01
EGF-like module-containing mucin-like hormone receptor-like 2	4.6E-01	9.3E-01
Tumor necrosis factor receptor superfamily member 19L	4.6E-01	9.3E-01
C-C motif chemokine 23	4.7E-01	9.3E-01
Interleukin-1 alpha	4.7E-01	9.3E-01
Complement C3b	4.8E-01	9.3E-01
Complement C3d fragment	4.9E-01	9.3E-01
Lymphocyte antigen 86	5.1E-01	9.3E-01
Lysozyme C	5.1E-01	9.3E-01
C-type lectin domain family 7 member A	5.1E-01	9.3E-01
Interleukin-23	5.2E-01	9.3E-01
Tumor necrosis factor ligand superfamily member 4	5.3E-01	9.3E-01

Phosphatidylinositol 4,5-bisphosphate 3-kinase catalytic subunit gamma isoform	5.3E-01	9.3E-01
C-C motif chemokine 18	5.3E-01	9.3E-01
Insulin-like growth factor-binding protein 4	5.7E-01	9.6E-01
Protein kinase C zeta type	5.8E-01	9.6E-01
Interleukin-1 beta	5.8E-01	9.6E-01
Complement C3b, inactivated	5.8E-01	9.6E-01
C-C motif chemokine 25	5.8E-01	9.6E-01
Bone morphogenetic protein 6	5.9E-01	9.6E-01
Tumor necrosis factor	6.0E-01	9.6E-01
Interleukin-10 receptor subunit beta	6.2E-01	9.6E-01
Mast/stem cell growth factor receptor Kit	6.3E-01	9.6E-01
Tumor necrosis factor receptor superfamily member 1B	6.4E-01	9.6E-01
Interleukin-1 Receptor accessory protein	6.4E-01	9.6E-01
C-X-C motif chemokine 5	6.4E-01	9.6E-01
Group IIE secretory phospholipase A2	6.4E-01	9.6E-01
C-C motif chemokine 7	6.5E-01	9.6E-01
Interleukin-17D	6.7E-01	9.6E-01
C-X-C motif chemokine 11	6.9E-01	9.6E-01
Tumor necrosis factor receptor superfamily member 4	6.9E-01	9.6E-01
Interleukin-18 receptor accessory protein	6.9E-01	9.6E-01
Advanced glycosylation end product-specific receptor, soluble	6.9E-01	9.6E-01
Protein DJ-1	7.1E-01	9.6E-01
Transforming growth factor beta-1	7.2E-01	9.6E-01
C-C motif chemokine 14	7.2E-01	9.6E-01
Interleukin-2 receptor subunit alpha	7.3E-01	9.6E-01
Carbohydrate sulfotransferase 2	7.4E-01	9.6E-01
CD5 antigen-like	7.5E-01	9.6E-01
Ck-beta-8-1	7.6E-01	9.6E-01
Tumor necrosis factor receptor superfamily member 10A	7.7E-01	9.6E-01
Calcium/calmodulin-dependent protein kinase type 1D	7.8E-01	9.6E-01
Interleukin-22	7.8E-01	9.6E-01
Interleukin-13	7.8E-01	9.6E-01
Interleukin-23 receptor	7.9E-01	9.6E-01
Macrophage migration inhibitory factor	7.9E-01	9.6E-01
Macrophage colony-stimulating factor 1	7.9E-01	9.6E-01
C-X-C motif chemokine 13	7.9E-01	9.6E-01
Interleukin-17A	8.1E-01	9.6E-01
Toll-like receptor 2	8.1E-01	9.6E-01
Allograft inflammatory factor 1	8.2E-01	9.6E-01
Oxidized low-density lipoprotein receptor 1	8.4E-01	9.6E-01
Tumor necrosis factor receptor superfamily member 9	8.6E-01	9.6E-01

Phosphatidylinositol 4,5-bisphosphate 3-kinase catalytic subunit alpha isoform:Phosphatidylinositol 3-kinase regulatory subunit alpha complex	8.6E-01	9.6E-01
E-Selectin	8.6E-01	9.6E-01
Tumor necrosis factor receptor superfamily member 25	8.7E-01	9.6E-01
Interleukin-1 receptor-like 2	8.7E-01	9.6E-01
C5a anaphylatoxin	8.7E-01	9.6E-01
C-C motif chemokine 22	8.8E-01	9.6E-01
Peroxiredoxin-5, mitochondrial	8.8E-01	9.6E-01
Natural cytotoxicity triggering receptor 3	8.8E-01	9.6E-01
Complement C3	9.0E-01	9.6E-01
Annexin A1	9.0E-01	9.6E-01
C-C motif chemokine 3	9.0E-01	9.6E-01
Complement C5	9.1E-01	9.6E-01
PSA:alpha-1-antichymotrypsin complex	9.1E-01	9.6E-01
Tumor necrosis factor-inducible gene 6 protein	9.1E-01	9.6E-01
C-C motif chemokine 13	9.2E-01	9.6E-01
Interleukin-27	9.3E-01	9.6E-01
Hepatitis A virus cellular receptor 2	9.4E-01	9.6E-01
Interleukin-8	9.4E-01	9.6E-01
C-C motif chemokine 16	9.5E-01	9.6E-01
Tumor necrosis factor receptor superfamily member 6B	9.5E-01	9.6E-01
Interleukin-17B	9.5E-01	9.6E-01
P-Selectin	9.7E-01	9.7E-01
